# Effects of carotid artery stenting on cognitive function in patients with mild cognitive impairment and carotid stenosis

**DOI:** 10.3892/etm.2013.954

**Published:** 2013-02-08

**Authors:** YONG CHENG, YAN JIANG WANG, JIA CHUAN YAN, RUI ZHOU, HUA DONG ZHOU

**Affiliations:** Department of Neurology and Center for Clinical Neuroscience, Daping Hospital, Third Military Medical University, Daping, Chongqing 400042, P.R. China

**Keywords:** carotid artery stenting, mild cognitive impairment, neuropsychological examinations, vascular risk factors, rapid verbal retrieval, Activities of Daily Living

## Abstract

Carotid stenosis is known to be an independent risk factor in the transformation process of mild cognitive impairment (MCI) to dementia and is treated by carotid artery stenting (CAS); however, the effects of CAS on cognitive function are unclear. In this study, 240 patients were prospectively assigned to a CAS or control group according to patient preference and underwent detailed neuropsychological examinations (NPEs) before and 6 months after treatment. Cerebral perfusion was assessed with computed tomography perfusion (CTP). Among the 240 patients included in the study, 208 patients completed NPEs at baseline and 6 months after therapy. The patients in the two groups did not differ with regard to baseline characteristics, educational level, vascular risk factors (VRFs) and NPEs prior to therapy. Significant improvements in the Mini-Mental State Examination (MMSE; before, 24.6±1.7 vs. after, 24.8±1.9; P=0.016), Montreal Cognitive Assessment (MOCA; before, 23.7±1.7 vs. after, 24.1±2.0; P=0.006), Fuld Object Memory Evaluation (FOME; before, 13.8±2.2 vs. after, 14.0±2.3; P=0.031) and Wechsler Adult Intelligence Scale-digital span (WAIS-DS; before, 6.7±2.1 vs. after, 6.9±2.3; P=0.040) were observed in the CAS group; however, improvements were not observed in the control group. Of the 84 patients in the CAS group who received CTP follow-up, 72 (86%) presented improvements in ipsilateral brain perfusion 6 months after the procedure; however, no improvement was observed in the control group. Close correlations were identified between the change in perfusion and the change in MMSE (r=0.575) and MOCA (r=0.574). CAS improves global cognitive function in patients with carotid stenosis and MCI and the improvement of cognition is closely related to the improvement of cerebral perfusion.

## Introduction

Mild cognitive impairment (MCI) is currently considered an early stage of dementia, which has no effective treatment. Reducing the progression of cognitive decline at the MCI stage may be an important strategy for preventing conversion to dementia (1). Carotid stenosis is known to be an independent risk factor in the transformation process of MCI to dementia (1–3). Cerebral perfusion deficiency caused by hemodynamic changes and cerebral emboli plays two key roles in this process (4–6). Cerebral emboli and hypoperfusion are ameliorated by angioplasty (7,8). Carotid artery stenting (CAS) has been shown to prevent the occurrence of strokes safely and effectively in multi-center studies (9,10); however, the effects of CAS on cognitive outcome in patients with carotid artery stenosis are controversial (11,12). A number of factors may lead to the variation in cognitive responses observed in the clinic, including differences in baseline cerebral perfusion status, detrimental effects on procedural emboli, temporary flow interruption and the beneficial effect of improved cerebral hemodynamics.

In this prospective study, we aimed to investigate the effect of CAS on neurocognitive function in patients with carotid stenosis and MCI.

## Patients and methods

### Subjects

A total of 240 inpatients with carotid stenosis and MCI were consecutively selected from the Department of Neurology, Daping Hospital, Chongqing from January 2008 to January 2011. They were assigned to a treatment group (CAS + drugs therapy, 167 cases) or a control group (simple drug therapy, 73 cases) according to patient preference. Eligibility requirements were: i) patients aged 55 years and older; ii) patients with symptomatic carotid stenosis >50% or asymptomatic carotid stenosis >70%, measured according to the North American Symptomatic Carotid Endarterectomy Trial (NASCET) criteria or its noninvasive equivalent (13); and iii) patients who were diagnosed with MCI. Exclusion criteria were: i) evidence of other significant stenosis (>50%) in the major arteries of the head or neck; ii) evidence of an acute cerebral infarction requiring emergency thrombolysis and/or emergency stent placement; iii) patients who had experienced a recent stroke (within 4 weeks, given the potential impact on cognitive function); iv) a history of previous subarachnoid or cerebral hemorrhage; v) a concomitant neurological disorder potentially affecting cognitive function, including severe Parkinson’s disease; vi) being unable to comply with the study assessment; vii) a mental illness or a score on the Hamilton Depression Rating Scale (HDRS) >17; viii) drug abuse; and ix) moving away or declining to participate.

### Protocol approvals and patient consent

This study was approved by the Institutional Review Board of the Third Military Medical University and all subjects and their care-givers provided informed consent. Registration number: ChiCTR-ONRC-12001879.

### Baseline data

At inclusion, data were collected on presenting symptoms, demographic characteristics and vascular risk factors (VRFs). Demographic data was composed of age, gender and educational level (lower educational level refers to an education time ≤6 years; higher educational level refers to an education time >6 years). The VRFs included hypertension, diabetes, hyperlipidemia, prior ischemic event, coronary artery disease, atrial fibrillation, current smoking habit and daily alcohol consumpton. The severity of carotid stenosis was grouped into moderate stenosis (51–69%) and severe stenosis (≥70%) by digital subtraction angiography (DSA) or computed tomography (CT) angiograms according to the NASCET method (13). The score on the National Institutes of Health Stroke Scale (NIHSS) (14) was assessed at baseline and at 1 day and 6 months after the procedure.

### Neuropsychological examinations (NPEs)

Cognition was assessed in the week preceding the procedure and 6 months after the procedure. NPEs were performed by two trained clinical neuropsychologists, who were blind to the outcome of the treatment. Mini-Mental State Examination (MMSE) and the Barthel Index of Activities of Daily Living (ADL), which were validated previously in elderly Chinese individuals (15,16), were used. The subjects with an abnormal MMSE score were assessed with HDRS to measure emotional status (17). Subsequently, a set of neuropsychological tests were applied, including Montreal Cognitive Assessment (MOCA), which identifies substantially more cognitive abnormalities following a transient ischemic attack (TIA) and stroke than the MMSE, to identify deficits in executive function, attention and delayed recall (18); Fuld Object Memory Evaluation (FOME) to detect extensive cognitive dysfunction mainly composed of memory (19); rapid verbal retrieval (RVR) to detect the function of semantic memory (20); and Wechsler Adult Intelligence Scale (WAIS) to evaluate immediate memory and function of graphical recognition (21).

### Diagnosis of MCI

The clinical diagnosis of MCI was conducted according to the established Petersen criteria (22), including: i) subjective complaint of memory deficits; ii) abnormal memory functioning for age [tests claim 1.5 standard deviation (SD) below normative values]; iii) absence of dementia according to the diagnostic examination [MMSE ≥24 in subjects with higher educational level; MMSE ≥20 in subjects with lower educational level; Clinical Dementia Rating (CDR) ≤0.5]; and iv) normal everyday functioning on ADL (<40). Subjects with depressive disorder were excluded (23).

### Treatment process and clinical follow-up

CAS was performed in the week after the patients were assigned to the treatment group, by routine use of an umbrella stent. Technical success was defined as implantation of a stent with a residual stenosis ≤30%; however, for patients with stenosis >90%, to reduce the risk of high perfusion syndrome postoperatively (24), residual stenosis was extended to ∼60%. Aspirin and clopidogrel were continued for 3 days before the procedure until 6 months after successful intervention. Patients in the control group were treated with the same oral medication as the treatment group. VRFs were carefully controlled in the two groups by management of blood pressure and blood glucose, as well as use of statins. Complete neurologic examinations were performed by an independent neurologist before, 1 week after and 6 months after treatment. Restenosis (restenosis rate >50%), ipsilateral ischemic events, neurologic sequelae, intracranial hemorrhages and mortalities were recorded. Follow-up clinical and ultra-sound examinations were scheduled at 6 months after therapy.

### Computed tomography perfusion (CTP)

**Figure 1:**
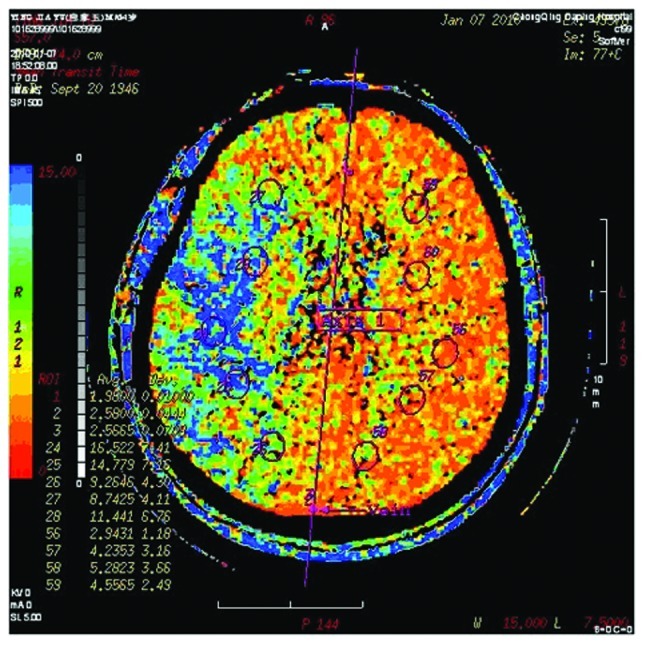
Brain perfusion computed tomography prior to intervention.

**Figure 2:**
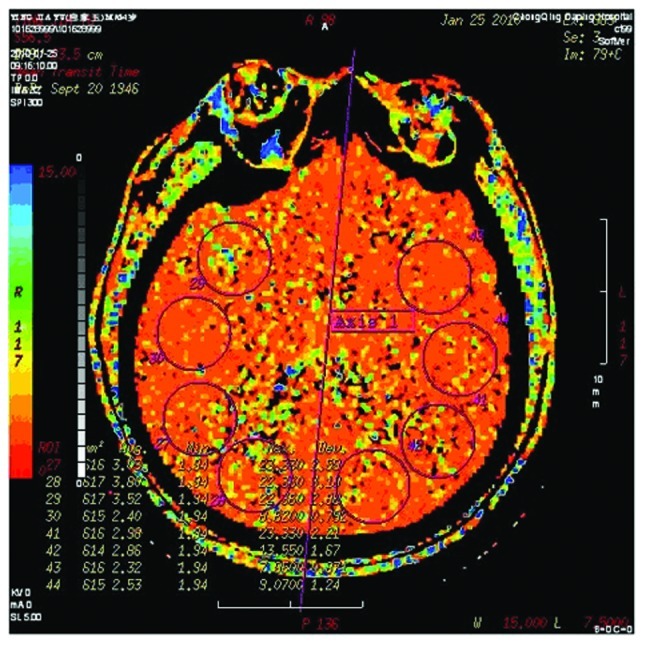
Three weeks after right carotid stenting, ipsilateral hemisphere perfusion was improved.

Brain CTP and CT angiography using a LightSpeed VCT 64-slice Scanner (GE Healthcare, Milwaukee, WI, USA) were scheduled in before and 3 weeks after treatment. Assessment of cerebral perfusion (at inclusion and follow-up) was performed by two independent investigators who were unaware of the clinical and angio-graphic outcomes. CTP data were analyzed on an advanced workstation (Advantage 4.2, GE Healthcare). Cerebral blood volume (CBV), cerebral blood flow (CBF), time to peak (TP) and mean transit time (MTT) were calculated. A grading system was used for qualitative assessment of the brain perfusion of the region of interest: 0, complete perfusion; 1, hypoperfusion with preserved CBV (lower CBF, delayed TP, increased MTT, decreased flow and normal or elevated CBV); and 2, hypoperfusion with decreased CBV. Improvement in brain perfusion after the procedure was defined as at least a 1 categorical number decrease in the region of interest according to the grading system (25). Figs. 1 and 2 are images of a patient who had significant improvement in ipsilateral brain perfusion following right carotid stenting.

### Statistical analysis

Continuous data are presented as mean ± SD. Discrete data are presented as counts and percentages. T-tests were used for continuous and normally distributed data and Chi-square analyses for comparing groups of categorical data. Paired continuous data were compared by the Wilcoxon signed rank sum test. Pearson’s correlation coefficients were used to assess the correlation between the change in brain perfusion and the changes in the results of neuropsychological tests. A two-sided P-value <0.05 was considered to indicate a statistically significant difference. Statistical analyses were performed using SPSS 18.0 for Windows (SPSS, Inc., Chicago, IL, USA).

## Results

Among the 240 patients registered in this study, 208 patients (144 in the CAS group and 64 in the control group) finished the NPEs and analysis of cognitive scores after treatment and 6 months of follow-up. The other 32 patients were excluded due to the following reasons: stenosis of 11 patients at the time of angiography did not conform to the enrollment criteria (<50% carotid stenosis in 4 cases and severe intracranial stenosis in 7 cases); 2 patients were unable to take the medicine on time at the proper dosage; 3 patients had severe dysphasia, hearing and visual impairment and worsening illness precluding evaluation; 3 patients refused NEP assessment; 2 patients in the CAS group crossed over to the control group and 1 patient in the control group crossed over to the CAS group and then left the study. Additionally, 4 patients (2 in the CAS group and 2 in the control group) were disabled due to complications and 6 patients (4 in the CAS group and 2 in the control group) refused to follow-up and left the study. The pretreatment CTP examinations were performed in 155 of the 218 patients (71%) and the post-treatment scan in 120 patients (58%).

Technical success was achieved in all patients in the CAS group. Following stent placement, the severity of carotid stenosis decreased to 21% (0–60%) vs. 68% (50–96%) preoperatively. The stenosis was left-sided in 62.5% of patients. In the 6 month follow-up, we observed stent restenosis in 4 patients (2.8%), ipsilateral cerebral infarction in 3 patients (2.1%) and ipsilateral TIA in 4 patients (2.8%). Of the 64 patients in the control group, 2 patients (3.1%) had ipsilateral cerebral infarction and 3 patients (4.7%) had ipsilateral TIA.

The patients in the two groups did not differ with regard to baseline characteristics, educational level, VRFs and NPEs prior to the procedure (Table I).

Table II shows neurocognitive and neurologic functions at baseline and after 6 months in the CAS and control groups. In the CAS group, we observed significant improvements in the MMSE (before, 24.6±1.7 vs. after, 24.8±1.9; P=0.016), MOCA (before, 23.7±1.7 vs. after, 24.1±2.0; P=0.006), FOME (before, 13.8±2.2 vs. after, 14.0±2.3; P=0.031) and WAIS-DS (before, 6.7±2.1 vs. after, 6.9±2.3; P=0.040). The change in MOCA was the most significant and RVR (before, 25.7±2.1 vs. after, 25.9±2.3; P=0.201) also exhibited an increasing trend. In comparison, all test parameters were decreased at follow-up in the control group, however the reductions were not statistically significant. NIHSS and ADL values were similar in the two groups at the 6 month follow-up compared with baseline results.

Of the 84 patients in the CAS group who received CTP follow-up, 72 (86%) demonstrated improvements in ipsilateral brain perfusion following the procedure; however, no improvements were identified in the control group. Table III shows the close correlations between the change in perfusion and the change in MMSE (r=0.575) and MOCA (r=0.574), as well as moderate correlations between the change in perfusion and the change in WIAS-DS (r=0.464), RVR (r=0.449) and FOME (r=0.375).

## Discussion

Approximately 4.6 million new patients worldwide are affected by Alzheimer’s disease (AD) every year, which has no effective treatment (26). Therefore, it is of great importance to recognize and treat the subjects at the MCI stage since it is an early stage of dementia (27) and is associated with an increased risk for progression to AD (10–15% per year), 10-fold more than in a normal population (22). Carotid artery stenosis is closely related to MCI and may be significant in the transition from MCI to dementia (1). Therefore, treatment of carotid artery stenosis at the MCI stage may be an important strategy for preventing and delaying the progression to dementia.

Mathiesen *et al* (28) reported that patients without a history of stroke who have carotid stenosis produce worse scores in a number of neuropsychological tests compared with those without carotid stenosis. Therefore, carotid stenosis plays a significant role in cognitive impairment. In a cohort study of 4,006 patients with asymptomatic carotid artery stenosis, Johnston *et al* (29) discovered that the thicker the carotid artery intima, the worse the cognitive function impairment. Additionally, cognitive dysfunction caused by severe left carotid artery (supplying the dominant cerebral hemisphere) stenosis is more serious and persistent. Rao (30) demonstrated that carotid stenosis may lead to frontal lobe damage. Current research suggests that carotid stenosis leading to cognitive impairment may be a result of chronic cerebral hypoperfusion, stroke, cerebral white matter lesions and potential vascular risk factors (5,16).

A multi-center, randomized, double-blind controlled study confirmed that carotid endarterectomy (CEA) has a positive effect on severe carotid stenosis (8). With the development of intervention materials and neuroimaging techniques, particularly the distal protection device for cerebral embolism, CAS is widely used in high-risk patients with carotid stenosis (31). The safety and effectiveness of CAS has been confirmed by clinical studies (9,32). Italy published the first CAS guide in 2006 and five associations in the United States also jointly issued a CAS guide in 2007 (33). Additional study has provided class III evidence that any difference between the effects of CAS and CEA on cognition at 6 months after revascularization is small (34), which made it possible for us to observe the cognitive function of patients with MCI and carotid artery stenosis by CAS. CEA is not widely applied in China. Among the 50 public hospitals that are developing the surgery, only 5 have a mature CEA technology center. As a result, the majority of patients in China preferentially select CAS, which is performed extensively. Therefore, the current study was set based only on the willingness of patients to join the CAS or control groups. A number of patients in the control group were finally treated with CAS after 6 months of follow-up.

Our study indicates that CAS increases the neuropsychological tests scores and/or psychomotor speed in MCI patients, although patients in the two groups experienced an ischemic event, stent restenosis (CAS group) and other complications in the six months of follow-up. Before the procedure, the MMSE, MOCA, FOME, WAIS-DS and RVR scores of the two groups were lower than normal. After the procedure, the MMSE, MOCA, FOME and WAIS-DS scores increased significantly in the CAS group. In addition, RVR demonstrated an improving trend. The NPE scores of the control group fell slightly after the 6 month follow-up; however, the reduction was not statistically significant. Therefore, this change was regarded as the gradual decline of cognition in MCI patients.

MMSE was selected as a test for its simple, reliable and large clinical application, which is sensitive to attention, repetition and language, but not abstract thinking, judgment, problem-solving and prediction. MOCA is based on visual-spatial implementation, naming and delayed memory. FOME focuses on delayed memory and recognition capability, while WAIS-DS focuses on evaluating immediate memory and the functioning of graphical recognition. The results of these two tests improved significantly following CAS. RVR is aimed at immediate memory and language fluency. The decline in RVR results was not evident at baseline, although the results improved slightly following the CAS procedure. The results of the above tests demonstrated that CAS delays the cognitive decline in patients with MCI.

To date, there has been no authoritative report on the incidence of MCI in patients with carotid artery stenosis and it is unknown which degree of carotid stenosis benefits from CAS. Particularly for a number of asymptomatic patients whose cognitive impairment may be subclinical and reversible, timely improved perfusion may lead to reversal of their cognitive impairment. In our prospective study, we identified that cerebral perfusion abnormalities are often observed in patients with severe carotid stenosis, whose cognitive scores improved more clearly following the procedure. Of the 84 patients who accepted CTP follow-up in the CAS group, 72 presented improvements in ipsilateral brain perfusion following the procedure and there were close correlations between the improvements in perfusion and improvements in cognitive score. This suggests that the perfusion improvements caused by vascular remodeling were the cause of cognitive benefits in patients with MCI.

In addition, several of the confounding factors associated with cognitive outcome following CAS may have been clarified in our study. Firstly, in the 6 month follow-up, we observed in-stent restenosis in 4 patients (2.8%), ipsilateral cerebral infarction in 3 patients (2.1%) and ipsilateral TIA in 4 patients (2.8%). Additionally, CAS carries the risk of subclinical micro-embolism (35–38), without surgical intervention in treatment group the likelihood of microembolism is smaller, and may have a negative effect on cognitive performance in the CAS group. Secondly, there was a natural course of cognitive decline in the 6 month follow-up in MCI patients, Nevertheless, the cognitive scores of the CAS group improved quite significantly following the procedure. Therefore, our results may underestimate the effects of vascular reconstruction on cognitive function improvement.

In the 6 month follow-up, we identified ipsilateral cerebral infarction in 2 patients (3.1%) and ipsilateral TIA in 3 patients (4.7%) in the control group. An ischemic event may affect cognitive function in patients to a certain extent, coupled with the natural decline of cognitive function in patients with MCI. The cognitive function of the control group declined; however, not significantly. We must take into account the VRFs, including hypertension and hyperlipidemia in patients, as well as the learning effect of completing NCEs twice.

There were no significant differences in baseline characteristics between the CAS and control groups. The same drug therapy was used in the two groups, so CAS was the only intervention. In contrast to previous studies that compared the preoperative and postoperative state of the same patient, we set up a control group to exclude the possibility of a learning effect (39). Therefore, the improvement in cognitive function in the CAS group is only explained by the restoration of cerebral perfusion and correction of hemispheric ischemia.

The current study has several limitations: i) the groups were selected according to the patients own preference, after being given a detailed explanation of the requirement for surgery and the risk of the surgery, rather than by random allocation; ii) the selection criteria was highly specific to reduce the effect of other factors, including posterior circulation and intracranial severe stenosis. Only patients with carotid artery stenosis and MCI were selected and patients with normal cognitive or dementia were excluded; iii) although significant improvements were observed in the patients of the CAS group, there were variations in individual patients and individual tests. The limitations of the cognitive tests in the Chinese population should be in reference to the average educational level or the date of validation of these cognitive tests in Mandarin. It is necessary to apply more specific neuropsychological tests to localize specific cortical functional zones in future studies; iv) the universality of our results is also relatively limited. It was not possible to clearly determine the duration of carotid stenosis in the two groups, particularly in asymptomatic patients. A longer duration of severe stenosis may potentially affect the reversibility of cognitive function.

Overall, our prospective study demonstrates significant improvements in cognition in patients with carotid stenosis and MCI 6 months after CAS and the improvement of cognition is closely related to the improvement of cerebral perfusion. More rigorous randomized controlled experiments and a longer follow-up duration are required to evaluate the long-term curative effect of CAS on the improvement of cognitive function in patients with CAS and MCI, as well as its role in delaying the progression of MCI to AD.

## Figures and Tables

**Table I:** Baseline patient characteristics (n=208).

Characteristics	CAS group (n=144)	Control group (n=64)	P-value
Age (years)	67.0±7.8	69.3±7.7	0.59
Females	48 (33.3)	22 (34.4)	0.88
Lower education level (≤6 years)	57 (39.6)	24 (37.5)	0.78
Hypertension	97 (67.4)	43 (65.6)	0.98
Diabetes mellitus	42 (29.2)	22 (34.4)	0.45
Hyperlipidemia	66 (45.8)	26 (40.6)	0.49
Prior ischemic event	79 (54.9)	36 (56.3)	0.85
Coronary artery disease	26 (18.1)	10 (15.6)	0.67
Atrial fibrillation	4 (2.8)	2 (3.1)	0.89
Smoking habit	34 (23.6)	14 (21.9)	0.78
Daily alcohol consumption	30 (20.8)	16 (25.0)	0.50
Severe carotid stenosis (>70%)	61 (42.4)	24 (37.5)	0.51
Left carotid stenosis	90 (62.5)	38 (59.4)	0.67
NIHSS score	0.72±1.16	0.69±1.15	0.87
ADL	23.0±2.7	23.1±2.8	0.88
MMSE	24.6±1.7	24.7±1.5	0.65
MOCA	23.7±1.7	23.8±1.5	0.60
FOME	13.8±2.2	14.2±2.3	0.17
RVR	25.7±2.1	26.0±1.9	0.45
WAIS-DS	6.7±2.1	6.8±2.0	0.86

Data are presented as mean ± standard deviation or n (%). CAS, carotid artery stenting; NIHSS, National Instiutes of Health Stroke Scale; ADL, Activities of Daily Living; MMSE, Mini-Mental State Examination; MOCA, Montreal Cognitive Assessment; FOME, Fuld Object Memory Evaluation; RVR, rapid verbal retrieval; WAIS-DS, Wechsler Adult Intelligence Scale-digital span.

**Table II:** Neuropsychologic test scores at baseline and follow-up.

	CAS group (n=144)			Control group (n=64)		
Test	Baseline	6 months after stenting	P-value	Baseline	6 months after medication	P-value
MMSE	24.6±1.7	24.8±1.9	0.016	24.7±1.5	24.5±1.6	0.137
MOCA	23.7±1.7	24.1±2.0	0.006	23.8±1.5	23.6±1.8	0.129
FOME	13.8±2.2	14.0±2.3	0.031	14.2±2.3	14.1±2.5	0.171
RVR	25.7±2.1	25.9±2.3	0.201	26.0±1.9	25.8±2.0	0.144
WAIS-DS	6.7±2.1	6.9±2.3	0.040	6.8±2.0	6.6±1.9	0.158
ADL	23.0±2.7	22.9±2.6	0.239	23.1±2.8	23.0±2.6	0.591
NIHSS	0.72±1.16	0.67±1.05	0.294	0.69±1.15	0.63±1.00	0.415

CAS, carotid artery stenting; NIHSS, National Institutes of Health Stroke Scale; ADL, Activities of Daily Living; MMSE, Mini-Mental State Examination; MOCA, Montreal Cognitive Assessment; FOME, Fuld Object Memory Evaluation; RVR, rapid verbal retrieval; WAIS-DS, Wechsler Adult Intelligence Scale-digital span. Values are presented as mean ± standard deviation (SD).

**Table III:** Pearson’s correlation coefficients between perfusion change and changes in NPE scores.

	MMSE change	MOCA change	FOME change	RVR change	WIAS-DS change
CTP change	0.575	0.574	0.375	0.449	0.464

MMSE, Mini-Mental State Examination; MOCA, Montreal Cognitive Assessment; FOME, Fuld Object Memory Evaluation; RVR, rapid verbal retrieval; WAIS-DS, Wechsler Adult Intelligence Scale-digital span; CTP, computed tomography perfusion; NPE, neuropsychological examination.

## Abbreviations:

CAS: carotid artery stenting;
MCI: mild cognitive impairment;
NPEs: neuropsychological examinations;
VRFs: vascular risk factors;
AD: Alzheimer’s disease;
NIHSS: National Institutes of Health Stroke Scale;
ADL: Activities of Daily Living;
MMSE: Mini-Mental State Examination;
MOCA: Montreal Cognitive Assessment;
FOME: Fuld Object Memory Evaluation;
RVR: rapid verbal retrieval;
WAIS-DS: Wechsler Adult Intelligence Scale-digital span
